# Sex-specific behavioral and monoaminergic network alterations following adolescent binge-like ethanol and WIN55,212-2 exposure under chronic nicotine in rats

**DOI:** 10.3389/fphar.2026.1798364

**Published:** 2026-04-10

**Authors:** Philippe De Deurwaerdère, Katie Haywood, Norbert Abela, Maurizio Casarrubea, Giuseppe Di Giovanni

**Affiliations:** 1 Université de Bordeaux, Bordeaux, France; 2 Centre National de la Recherche Scientifique, INCIA UMR 5287, Bordeaux, France; 3 Laboratory of Neurophysiology, Department of Physiology and Biochemistry, Faculty of Medicine and Surgery, University of Malta, Msida, Malta; 4 Laboratory of Behavioral Physiology, Department of Biomedicine, Neuroscience and Advanced Diagnostics (Bi.N.D.), Human Physiology Section “Giuseppe Pagano”, University of Palermo, Palermo, Italy; 5 Neuroscience Division, School of Biosciences, Cardiff University, Cardiff, United Kingdom; 6 College of Medicine, Korea University, Seoul, Republic of Korea; 7 Department of Medical and Surgical Sciences, University of Magna Graecia, Catanzaro, Italy

**Keywords:** allostatic plasticity, corticolimbic networks, emotional regulation, exploratory behavior, monoaminergic reorganization

## Abstract

**Introduction:**

Recreational drug use often begins during adolescence, a critical period of brain maturation characterized by heightened vulnerability to drug-induced neuroplastic changes. Although weekend consumption of alcohol and cannabis and nicotine is commonly perceived as relatively harmless, the long-term neurobiological consequences of such patterns remain poorly understood.

**Methods:**

In this study, we investigated the behavioral and neurochemical consequences of combined adolescent exposure to moderate doses of alcohol, cannabinoid receptor agonist WIN55,212-2, and nicotine in male and female Long-Evans rats. From postnatal day (P)30 to P60, animals received daily nicotine (0.3 mg/kg, i.p.) together with intermittent ethanol (3 g/kg, intragastric) and WIN55,212-2 (1.2 mg/kg, i.p.) administered twice weekly, modeling a weekend-like binge pattern of substance use. Behavioral assessments were conducted at baseline (P30), immediately after treatment (P60), and in early adulthood (P90) using the hole-board (HB) and elevated plus maze (EPM) tests. Monoamine levels were subsequently quantified *postmortem* in selected cortical, limbic, and midbrain regions involved in anxiety and reward processing and correlation analysis performed to explore relationships between neurochemical across these brain regions.

**Results:**

In the HB test, but not in the EPM, treated females exhibited behaviors consistent with increased anxiety-like responses and reduced exploratory drive, including reduced walking and head-dipping, increased grooming, and decreased climbing at P60, with some effects persisting at P90. In contrast, males were minimally affected. Neurochemical analyses revealed only modest changes in absolute serotonin (5-HT) levels in the prefrontal cortex (PFC), with no significant alterations in dopamine (DA) or noradrenaline (NA). However, correlation analyses uncovered a pronounced, sex-dependent reorganization of monoaminergic networks. Females showed increased functional coupling among DA, 5-HT, and NA pathways, particularly involving the PFC and amygdala, whereas males exhibited reduced dopaminergic coherence within the substantia nigra.

**Discussion:**

Together, these findings demonstrate that even moderate-low levels of adolescent polydrug exposure can produce long-lasting, sex-specific behavioral alterations, most prominently in females, alongside persistent remodeling of monoaminergic circuitry in both sexes. These results highlight the vulnerability of the adolescent brain to seemingly moderate recreational drug patterns and underscore the importance of considering sex-dependent neurobiological adaptations in assessing long-term neuropsychiatric risk.

## Introduction

Alcohol, cannabis, and nicotine, increasingly consumed through vaping (Becker and Rice, 2022), are the most commonly used psychoactive substances among adolescents worldwide, largely due to their social acceptability and accessibility (Moor et al., 2020; United Nations Office onD. C, 2020). The recent expansion of medical and recreational cannabis legalization has raised concerns about potential increases in adolescent cannabis use and polysubstance exposure, although current epidemiological evidence remains mixed (Hammond et al., 2020). Despite a decline after the COVID-19 pandemic (NIDA, 2024), adolescent substance use among adolescents remains a major public health concern because of its long-term consequences (Harper et al., 2023; Steinfeld and Torregrossa, 2023). Adolescence, the transitional period between childhood and adulthood (approximately 12-17 years of age), represents a critical developmental window characterized by extensive structural and functional reorganization of brain circuits involved in attention, reward processing, emotional regulation, and executive control (Yurgelun-Todd, 2007; Blankenstein et al., 2022; Reynolds et al., 2025). Consequently, exposure to alcohol, cannabis, and nicotine through cigarette smoking or vaping during this period may disrupt normal brain maturation and lead to long-term brain alteration, including decrease in hippocampal volume (Harper et al., 2023), together with behavioral and cognitive consequences (Luciana and Feldstein Ewing, 2015).

Females may be more vulnerable than males to the detrimental effects of substance use during adolescence (Heitzeg et al., 2018). Such sex differences have been reported for binge drinking (Squeglia et al., 2012; Pérez-García et al., 2022), cannabis use (Calakos et al., 2017; Cooper and Craft, 2018), tobacco dependence, or their co-abuse in both women (Cross et al., 2017; Harper et al., 2023) and female animals (Abela et al., 2023; Casarrubea et al., 2024). Considering the likely influence of gonadal steroids in sex differences (Becker and Hu, 2008; Maher et al., 2022), alcohol, cannabinoids, and nicotine exert convergent effects on central monoaminergic systems beyond their primary pharmacological targets (Pava and Woodward, 2012; Volkow and Blanco, 2023). The monoamines serotonin (5-HT), noradrenaline (NA), and dopamine (DA) originate from neuronal populations in the raphe nuclei, locus coeruleus, and ventral midbrain regions (substantia nigra: SN and ventral tegmental area: VTA) and project widely throughout the brain. These systems regulate mood, motivation, learning, and emotional behavior and are central to the pathophysiology of neuropsychiatric disorders (Di Giovanni et al., 2010; De Deurwaerdere et al., 2021). Chronic exposure to alcohol and other addictive substances induces significant adaptations in central monoaminergic systems. In alcohol use disorder (AUD), dysregulation of mesolimbic dopaminergic signaling contributes to altered reward processing and compulsive alcohol use (Koob, 2008; Volkow et al., 2016; Prajapati et al., 2020). Serotonergic alterations in cortical and limbic regions are linked to affective instability and relapse vulnerability (Müller and Homberg, 2015), while noradrenergic projections mediate stress-related aspects of addiction and withdrawal (Koob, 2008). Importantly, nicotine and cannabinoids similarly modulate these monoaminergic pathways, and combined exposure may produce convergent neuroadaptations across DA, 5-HT, and NA systems (Pava and Woodward, 2012; Prajapati et al., 2020; De Deurwaerdere et al., 2021). Cognitive processes are better represented by coordinated activity across distributed brain networks rather than by activity in isolated regions. Consistent with this view, polysubstance exposure has been associated with alterations in resting state functional connectivity (Squeglia et al., 2012; Wetherill et al., 2015), with evidence indicating that these network level disruptions may emerge as early as adolescence. These findings support the investigation of large-scale monoaminergic network reorganization following adolescent polydrug exposure. In rodents, monoaminergic function and regional interactions can, indeed, be studied through neurochemical profiling of monoamine content and turnover across brain regions (Fitoussi et al., 2013; Dellu-Hagedorn et al., 2017; Gros et al., 2021, Butler et al., 2025b), allowing the assessment of large-scale neurotransmitter connectivity potentially altered by polydrug exposure.

A pattern often perceived by youth as harmless is weekend consumption of a few alcoholic drinks (moderate binge-drinking) combined with moderate cannabis use in light smokers. This pattern has recently been modeled in preclinical research (Abela et al., 2023) and was shown to induce sex-dependent impairments in adult reward learning and goal-directed behavior, with more pronounced effects in females (Abela et al., 2023). Building on these findings, we hypothesized that the same polydrug paradigm would produce sex- and age-dependent behavioral alterations, including increased anxiety-like behavior, accompanied by region-specific changes in monoaminergic correlations.

To test this hypothesis, adolescent male and female Long-Evans rats were exposed to a polydrug regimen from P30 to P60 (Abela et al., 2023), receiving daily intraperitoneal (i.p.) injections of a low dose of nicotine (0.3 mg/kg, i.p.) (Matta et al., 2007), modelling habitual smoking/vaping. In addition, rats were administered twice-weekly intragastric ethanol (3 g/kg), to model moderate binge-like intoxication (Crabbe et al., 2011) together with the synthetic cannabinoid receptor agonist WIN55,212-2 (1.2 mg/kg, i.p.) (Compton et al., 1992; Fox et al., 2001) to mimic CB1 receptor activation associated with recreational cannabis use. The intermittent ethanol/WIN55,212-2 exposure was structured to reflect a “weekend” binge-like consumption pattern superimposed on chronic nicotine background exposure. Behavioral performance was assessed using the hole-board (HB) and elevated plus maze (EPM) tests at baseline (P30), and immediately after treatment (P60) and following a washout period of 30 days (P90), in order to evaluate exploratory and anxiety-related behaviors. To obtain a comprehensive assessment of large-scale monoaminergic connectivity, monoamine concentrations and turnover rates were measured in selected cortical, limbic, and midbrain regions *postmortem* (>P100) using high performance liquid chromatography with electrochemical detection (HPLC-ED) (De Deurwaerdère et al., 2022).

## Methods

### Subjects

Sample size was determined based on prior studies from our group employing the same behavioral tests. Twenty male and twenty female Long-Evans rats were bred and housed in the Animal Facility of the Department of Physiology and Biochemistry, University of Malta. The animals were housed in standard plastic cages (3-4 rats per cage) under controlled environmental conditions (12/12 h light/dark cycle, 22 °C ± 2 °C, 55% ± 10% humidity) with *ad libitum* access to food and water. Rats were separated by sex and randomly assigned to control or treatment groups, yielding four experimental groups in total. All procedures complied with European Directive 2010/63/EU and with the guidelines of the Animal Welfare Committee of the Faculty of Medicine and Surgery, University of Malta.

### Drugs

Nicotine hydrogen tartrate salt and WIN55,212-2 were purchased from Sigma-Aldrich (St. Louis, MO, USA). Nicotine was dissolved in 0.9% saline (1 mg/mL as the salt), and the pH was adjusted to 7.4 using NaOH and HCl. It was administered intraperitoneally (i.p.) at a dose of 0.3 mg/kg (free base). WIN55,212-2 was selected as a well-characterized synthetic CB1 receptor agonist that provides stable pharmacokinetics and consistent receptor activation, allowing a controlled and reproducible experimental modeling of cannabinoid exposure (Fox et al., 2001). WIN55,212-2 was prepared at a concentration of 0.6 mg/mL in a vehicle solution containing 5% polyethylene glycol (PEG), 5% polysorbate 20 (Tween-20), and 90% saline. For preparation, WIN55,212-2 was first dissolved in PEG and stored at −18 °C. On the day of use, the required volume was thawed at room temperature for 30 min, mixed with Tween-20 and saline, and vortexed. The solution was administered intraperitoneally at 1.2 mg/kg within 1 hour of preparation. Ethanol (3 g/kg, intragastric) was administered to model moderate binge-like intoxication, a dose widely used in rodent studies that produces blood alcohol concentrations comparable to those observed in human binge drinking paradigms (Crabbe et al., 2011). Adolescent rats display reduced behavioral sensitivity to ethanol compared with adults, often requiring relatively higher doses to elicit comparable intoxication-related effects; 3 g/kg represents the lowest dose reliably producing such effects (Chandler et al., 2022; Vetreno et al., 2023), corresponding to blood alcohol concentration of approximately 130 mg/kg (Matthews et al., 2023). Ethanol was prepared as a 25% (w/v) solution. Considering the density of pure ethanol (0.789 mg/mL), 32 mL of absolute ethanol was mixed with 100 mL of distilled water.

### Drug treatment procedure

Treatment began when rats reached P30 and continued for 30 consecutive days. Treated rats received daily i.p. injections of nicotine (0.3 mg/kg). On two consecutive days each week, they also received i.p. injections of WIN55,212-2 (1.2 mg/kg) and ethanol (3 g/kg) by gavage (per os, p.o.) (Abela et al., 2023).

**FIGURE 1:**
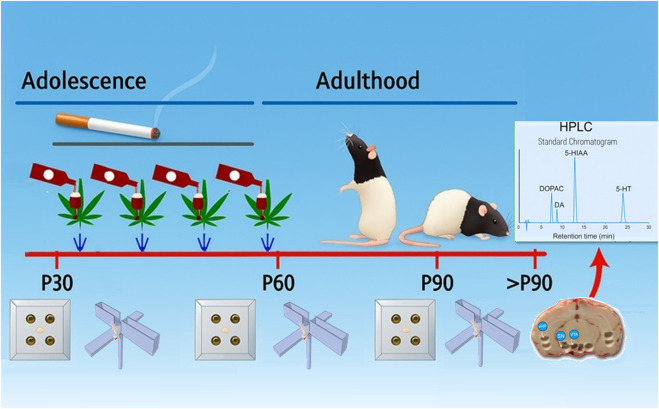
Experimental design. Male and female Long–Evans rats began treatment at postnatal day 30 (P30) and received binge-like exposure for 28 days (twice weekly) to alcohol (3 g/kg, p.o.) and the cannabinoid agonist WIN55,212-2 (1.2 mg/kg, i.p.), together with daily nicotine (0.3 mg/kg, i.p.). Behavioral assessments using the hole-board (HB) and elevated plus maze (EPM) tests were performed at P30, P60, and P90. Post-mortem neurochemical analyses of monoamines in brain regions implicated in decision-making and anxiety were conducted after P90 [modified from (Abela et al., 2023)].

The gavage was performed using a syringe fitted with a stainless-steel feeding needle equipped with a rubber tip to facilitate comfortable oral insertion. The needle was cleaned with 70% ethanol and dried between each use. The treatment timeline is illustrated in Figure 1. Control animals followed the same schedule and received corresponding vehicles: saline instead of nicotine, the PEG/Tween-20/saline solution instead of WIN55,212-2, and distilled water instead of ethanol.

### Behavioral tests

Behavioral recordings were conducted at P30, P60, and P90. Before testing, rats were transported to a dimly lit room in their home cages and allowed to acclimate for 30 min.

#### Hole-board (HB)

The HB apparatus consisted of a white Plexiglas platform (square shape) surrounded by three opaque walls and one transparent wall. Four holes (4 cm in diameter) were evenly spaced, one near each corner. The platform was elevated 5 cm above the surface. A video camera was positioned in front of the transparent wall at an angle of 45°, allowing full coverage of the arena. Each rat was placed in the center of the HB and allowed to explore for 10 min (Casarrubea et al., 2010; Casarrubea et al., 2023).

#### Elevated plus maze (EPM)

The EPM consisted of two open arms (without walls) and two closed arms (surrounded by opaque plastic walls), elevated 50 cm above the floor by metal supports. A video camera was positioned above the maze to capture all four arms. Each rat was placed in the center of the platform and allowed to explore for 5 min (Casarrubea et al., 2013; Cassar et al., 2022; Casarrubea et al., 2023; Prajapati et al., 2024).

After each session, the apparatuses were cleaned with 70% ethanol to remove scent traces and feces. All behavioral sessions were conducted between 09:00 and 18:00 h.

### Behavioral analysis

**FIGURE 2:**
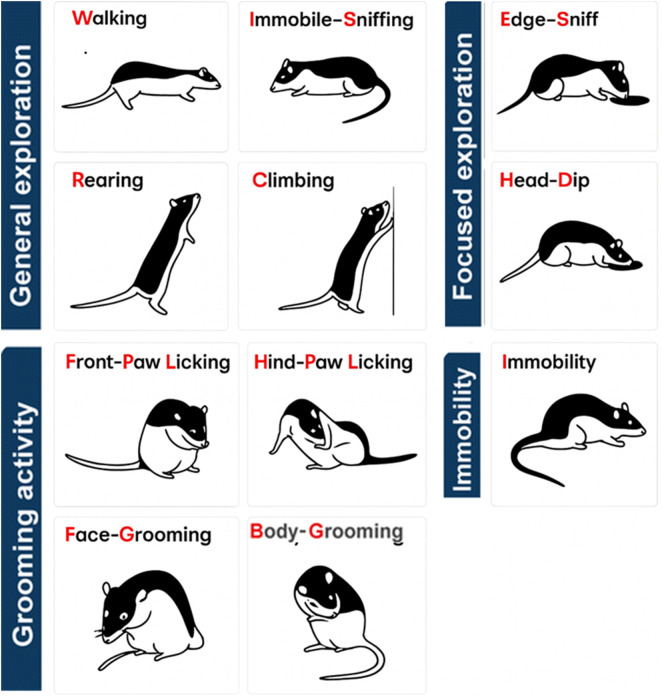
Ethogram. Ethogram illustrating the behavioral repertoire of rats in the hole-board apparatus. Walking (Wa): the rat moves around the arena while actively sniffing the surroundings; Immobile Sniffing (IS): the rat remains stationary while exploring the environment through olfaction; Climbing (Cl): the rat stands upright with its forepaws resting against the Plexiglas wall; Rearing (Re): the rat stands on its hind limbs without support from the walls; Edge Sniffing (ES): the rat investigates the rim of one of the four holes by sniffing; Head Dipping (HD): the rat inserts its head into one of the holes. Front-Paw Licking (FPL): the rat licks or grooms its forepaws; Hind-Paw Licking (HPL): the rat licks or grooms its hind paws; Face Grooming (FG): the rat cleans its face (ears, mouth, vibrissae, and eyes) using rapid circular forepaw movements; Body Grooming (BG): the rat licks and smooths its body fur using quick strokes of its incisors. Immobility (Imm): the rat remains motionless, maintaining a stable posture without producing any visible movements [modified from (Casarrubea et al., 2015)].

HB behaviors were analyzed using The Observer XT software (Noldus Information Technology). Behavioral categories, defined according to the ethogram (see Figure 2), included locomotor, exploratory, and grooming activities. Mean frequencies and durations were calculated for each behavioral element. For the EPM, ImageJ software was used to automatically quantify the number of entries into open and closed arms and the time spent in each. These data were used to calculate mean frequencies and durations for open- and closed-arm exploration.

### Selection of tissues

Around postnatal day 100 (>P90), rats were sacrificed, and their brains were rapidly removed, frozen in dry ice, and stored at −80 °C. Tissue dissection was performed using a cryostat set to −24 °C, in order to prevent tissue degradation. The brain was positioned so that punching began at the frontal section. Unilateral tissue punches were collected from the following areas for their functions: the prefrontal cortex (PFC) and cingulate cortex (CC) involved in executive control (Miller and Cohen, 2001); nucleus accumbens and striatum (NAc and STR) implicated reward processing (Haber and Knutson, 2010); thalamus (TH) serving as an integrative relay (Sherman and Guillery, 2013); amygdala (Am) regulating emotional salience (Janak and Tye, 2015); dorsal and ventral hippocampus (dHP and vHP) contributing to memory and affect (Fanselow and Dong, 2010); and substantia nigra (SN) providing dopaminergic output (Björklund and Dunnett, 2007). Each sample was collected in pre-weighed, labeled microtubes, weighed, and stored at −80 °C until analysis (Dellu-Hagedorn et al., 2017).

### Neurochemical analysis

Monoamine and metabolite concentrations were quantified using high-performance HPLC-ED. Tissue samples were homogenized in 100 μL of 0.1 M perchloric acid (HClO_4_) using sonication and centrifuged at 13,000 rpm for 30 min at 4 °C. Supernatants (10 μL) were injected manually (Rheodyne 7225i; C.I.L.-Cluzeau) into an Equisil BDS C18 column (150 × 4.6 mm, 5 μm; C.I.L.-Cluzeau, Sainte-Foy-La-Grande, France).

The mobile phase consisted of 70 mM NaH_2_PO_4_, 0.1 mM EDTA, 2 mM sodium octane sulfonate, 100 μL/L triethylamine, and 7% methanol, adjusted to pH 4.2 with orthophosphoric acid. The flow rate was 1.3 mL/min (LC20-AD pump, Shimadzu, France). The mobile phase was filtered through a 0.22 μm Millipore filter before use. Monoamines (NA, DA, 5-HT) and metabolites [5-hydroxy indoleacetic acid (5-HIAA), 3,4-dihydroxyphenylacetic acid (DOPAC)] were detected using a coulometric detector (Coulochem II, ESA, Paris, France) connected to an analytical cell (Model 5011). Electrode potentials were set at −270 mV (reduction) and +350 mV (oxidation). Data were acquired with Azur software (Alphamos, Toulouse, France). Standard solutions for each analyte were injected daily to verify chromatographic and electrochemical performance.

### Statistical analysis

Behavioral data from the hole-board (HB) and elevated plus maze (EPM) tests were analyzed using mixed-design ANOVA. For HB and EPM parameters, age (P30, P60, P90) was treated as a within-subject factor, while treatment and sex were considered between-subject factors. When significant main effects or interactions were detected, Fisher’s PLSD test was applied for *post hoc* multiple comparisons (n = 10 per group).

Monoamine and metabolite concentrations were expressed as pg/mg tissue weight. Turnover indices were calculated as the ratios DOPAC/DA and 5-HIAA/5-HT. Outliers were excluded if values exceeded the mean ±2 standard deviations (Fitoussi et al., 2013). Statistical analysis was performed using two-way ANOVA with treatment and sex as main factors. When significant interactions were found, Fisher’s PLSD test was used for multiple comparisons. For non-parametric data (metabolites and/or ratios), Kruskal–Wallis followed by Dunn’s *post hoc* test was applied. Correlation analyses were conducted using the Bravais–Pearson correlation coefficient (r) for monoamine, metabolite, and turnover data (n = 10 per group before outlier removal).

Data were expressed as mean ± SEM and considered significant at p < 0.05.

## Results

### Effect of binge-like polydrug exposure on female and male rat behaviors in the hole-board (HB)

**FIGURE 3:**
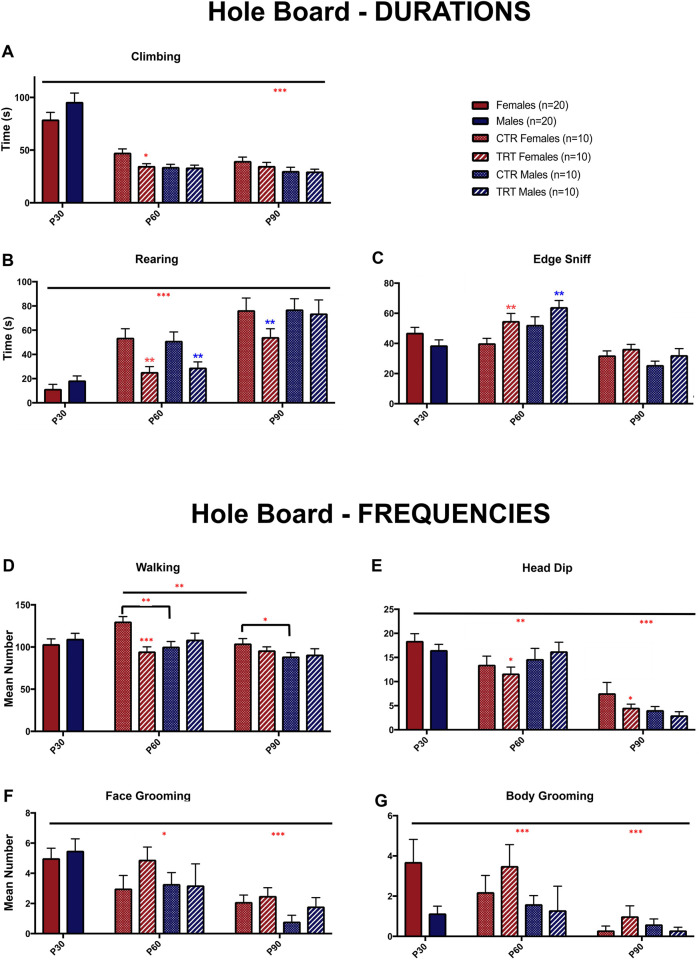
Effects of adolescent binge-like alcohol and cannabinoid exposure (2 days/week) during chronic nicotine treatment (P30–P60) on behavior in the hole-board (HB) test in male and female rats. (**A–C**) Duration and (**D–G**) frequency of distinct behaviors measured at postnatal days (P) 30, 60, and 90 in control (CTR) and treated (TRT) groups. Treatment consisted of daily nicotine (0.3 mg/kg, i.p.) from P30 for 28 consecutive days, with WIN55,212-2 (1.2 mg/kg, i.p.) and ethanol (3 g/kg, p.o.) administered twice weekly. Values are expressed as mean ± SEM (n = 10 per group: CTR females, TRT females, CTR males, TRT males). *p < 0.05, **p < 0.01, ***p < 0.001 (three-way ANOVA followed by Fisher’s PLSD test). See text for details.

In the HB test, adolescent exposure to alcohol and cannabinoid receptor agonist WIN55,212-2 in a binge-like pattern (2 days/week), combined with daily nicotine administration from P30 to P60 (Figure 1; Supplementary Table S1) produced significant sex- and age-dependent alterations in behavioral parameters (Figure 2), both acutely at P60 and after a 30-day washout period in adulthood (P90) (Figure 3).

For duration measures (Figure 3), climbing duration was affected by treatment (p < 0.05). Two-way ANOVA also revealed a significant age × sex interaction (p < 0.005), with climbing duration differing between P30 and P90 animals, such that P90 rats exhibited shorter climbing times. Post hoc analyses revealed a reduction in climbing duration in females at P60 compared with control (p < 0.01) (Figure 3A).

Rearing duration showed main effects of treatment (p < 0.005) and age (p < 0.0001), with a progressive increase across development (all p < 0.001). Post hoc analyses revealed a reduction in rearing duration in females and males at P60 (p < 0.01) and females at P90 (p < 0.01), compared with controls (Figure 3B).

Edge-sniffing duration was significantly influenced by treatment (p < 0.01) and age (p < 0.001), with a significant age × sex interaction (p < 0.01), suggesting sex-specific developmental trajectories. At P60, a significant difference was observed between treatment types in both sexes (p < 0.01), with treated rats displaying a greater duration of edge sniffing (Figure 3C).

For frequency measures (Figure 3), walking frequency showed a main effect of age (p < 0.005), a significant treatment × sex interaction (p < 0.001), and age × treatment (p < 0.05) and age × treatment × sex (p < 0.05) interactions. Post hoc analyses revealed that treatment decreased walking frequency in females at P60 (p < 0.001). Developmental comparisons showed significant age- and sex-related decreases in walking frequency from P60 to P90 in control females (p < 0.001) and in male vs. female controls at P60 (p < 0.01) and P90 (p < 0.05) (Figure 3D).

Head dip frequency showed a main effect of treatment (p < 0.05) and age (p < 0.001), as well as an age × sex interaction (p < 0.05). Post hoc analyses indicated that treatment reduced head-dipping frequency in females at P60 and P90 (both p < 0.05), whereas no significant treatment effect was observed in males. Head dipping decreased with age, with differences between P30 and P90 (p < 0.001), and P60 and P90 (p < 0.01) in both sexes, while differences between P30 and P60 (p < 0.005) were observed only in female (Figure 3D).

Face grooming frequency showed a significant main effect of age (p < 0.001), decreasing progressively from P30 to P90 (p < 0.01). Post hoc analyses revealed a significant increase in grooming in treated females at P60 (p < 0.01), whereas no treatment differences were observed at P30 prior to exposure and P90 (Figure 3F).

Body grooming frequency showed effects of sex (p < 0.05) and age (p < 0.005). By P90, body grooming frequency declined across groups, indicating a developmental reduction rather than a persistent treatment effect (P30 vs. P90 and P60 vs. P90 p < 0.005) (Figure 3F).

Taken together, these findings indicate that adolescent exposure of rats to polydrug administration produces selective and partly long-lasting age- and sex-dependent effects on spontaneous behavior. Development exerted a modulatory influence, with several exploratory behaviors (e.g., head-dipping and grooming) declining from adolescence to adulthood. Superimposed on these maturational changes, polydrug exposure induced a predominantly female-specific behavioral profile, characterized by reduced exploratory drive (walking and head-dipping frequency, as well as rearing and climbing duration) and increased edge-sniffing behavior at P60, with partial persistence into adulthood (notably reduced head-dipping frequency and rearing duration). These sex-dependent effects suggest greater vulnerability of females to adolescent polydrug exposure, possibly reflecting differences in the maturation of corticolimbic circuits regulating motivation, emotional reactivity, and behavioral flexibility.

### Effect of binge-like polydrug exposure on female and male rats in the elevated plus maze (EPM)

**FIGURE 4:**
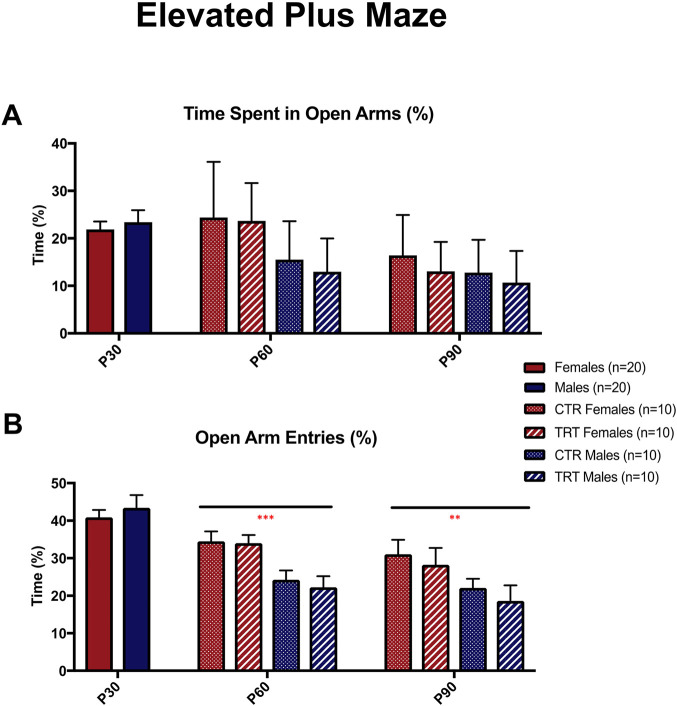
Effect of adolescent alcohol and cannabis exposure in a binge-like pattern (2 days/week) on male and female subjects treated daily with nicotine (P30–P60) in the elevated plus maze (EPM) test. **(A)** Time spent in the open arms (%) and **(B)** percentage of open arm entries at postnatal days (P)30, P60, and P90 in control (CTR) and treated (TRT) rats. Treatment consisted of daily nicotine (1 mg/kg, i.p.) from P30 for 28 days, with WIN55,212-2 (1.2 mg/kg, i.p.) and ethanol (3 g/kg, p.o.) administered twice weekly. Females exhibited greater open arm exploration and occupancy than males, reflecting reduced anxiety-like behavior. A significant effect of age was observed, with increased open arm activity from P30 to P90, indicating a developmental reduction in anxiety. No significant differences in total distance traveled were detected (data not shown), suggesting unchanged locomotor activity. Values are expressed as mean ± SEM. n = 10 per group (CTR females, TRT females, CTR males, TRT males). **p < 0.01, ***p < 0.001 (three-way ANOVA followed by Fisher’s PLSD test).

As shown in Figure 4A (and Supplementary Table S1), the analysis of open arm stay time (%) revealed a significant main effect of age (p < 0.001). Post hoc Fisher’s PLSD tests indicated a progressive increase in time spent in the open arms across development, with significant differences between P30 and P60 (p < 0.01), P30 and P90 (p < 0.001), and P60 and P90 (p < 0.001). No significant main effects of sex or treatment were detected for this parameter.

For open arm entries (%) (Figure 4B), significant main effects of sex (p < 0.05) and age (p < 0.001) were observed, along with a significant age × treatment interaction (p < 0.01). Post hoc tests revealed that females made more open arm entries than males at P60 (p < 0.001) and P90 (p < 0.05), whereas no difference was found at P30 (p = 0.5401). However, simple comparisons did not reveal consistent differences between treated and control animals within individual age groups.

Total distance travelled (data not shown) did not differ significantly among treatment (p = 0.6145), sex (p = 0.3892), or age groups (p = 0.2768), indicating that the observed changes in open arm behavior were not due to alterations in overall locomotor activity.

No clear treatment-induced alterations were detected in the EPM, in contrast to the HB test. This apparent discrepancy likely reflects differences in the behavioral dimensions captured by the two paradigms and further support a complementary battery of tests for a more comprehensive assessment of anxiety (Acikgoz et al., 2022).

Instead, these findings demonstrate a robust age- and sex-dependent modulation of anxiety-like behavior. Females showed greater open arm exploration and occupancy than males, suggesting lower anxiety levels.

### Effect of binge-like polydrug exposure on monoamines of female and male rats

#### Quantitative assessment

**FIGURE 5:**
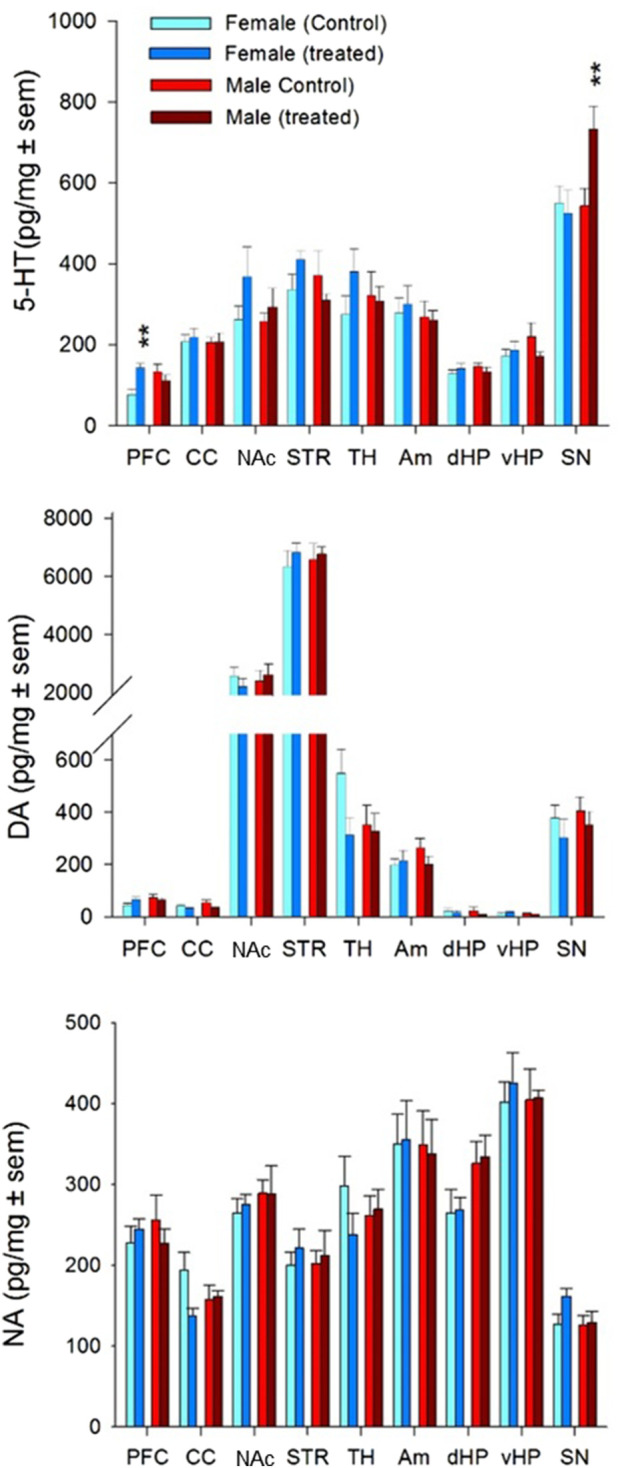
Effect of adolescent alcohol and cannabinoid agonist exposure in a binge-like pattern combined with daily with nicotine administration (P30-P60) on tissue monoamine levels in different brain regions of male and female of Long-Evans rats. Monoamine concentrations were measured at > P90 and are expressed in pg/mg ± SEM for serotonin (5-HT), dopamine (DA), and noradrenaline (NA) across the indicated brain regions. Treatment effects (Treated vs. Controls) were analyzed using a two-factor ANOVA design (Treatment x Sex) (see Supplementary Table S2). PFC, prefrontal cortex; CC, cingulate cortex; NAc, nucleus accumbens; STR, striatum; TH, thalamus; Am, amygdala; dHP, dorsal hippocampus; vHP, ventral hippocampus; SN, substantia nigra. *p < 0.05; **p < 0.01 (two-way ANOVA followed by Fisher’s PLSD test).

The effects of binge-like polydrug exposure on brain monoamine neurochemistry and metabolism are presented in Figure 5 (neurotransmitters) and Supplementary Table S2 (metabolites and turnover ratios). The Supplementary Table S3 summarizes the results of the statistical analyses.

Overall, the tissue concentrations of 5-HT, DA, and NA were comparable between female and male rats across the sampled brain regions, including the PFC, CC, NAc, STR, TH, Am, dHP, vHP, and SN. The only sex difference observed under basal conditions was a lower 5-HT content in the PFC of females compared to males.

The interaction between sex and binge treatment did not significantly affect the DA or NA systems. However, it did alter the 5-HT system in a region-specific and sex-dependent manner. In females, 5-HT and its primary metabolite 5-HIAA were significantly increased in the PFC and 5-HIAA in vHP following binge-like exposure to alcohol and cannabinoids compared with their respective controls. In males, similar increases in 5-HT levels were observed in the SN. Furthermore, the 5-HIAA/5-HT ratio, an indirect index of serotonergic turnover, was significantly elevated in the vHP of treated females relative to control females (Supplementary Table S3), suggesting enhanced serotonergic activity in this region.

#### Qualitative assessment

**FIGURE 6:**
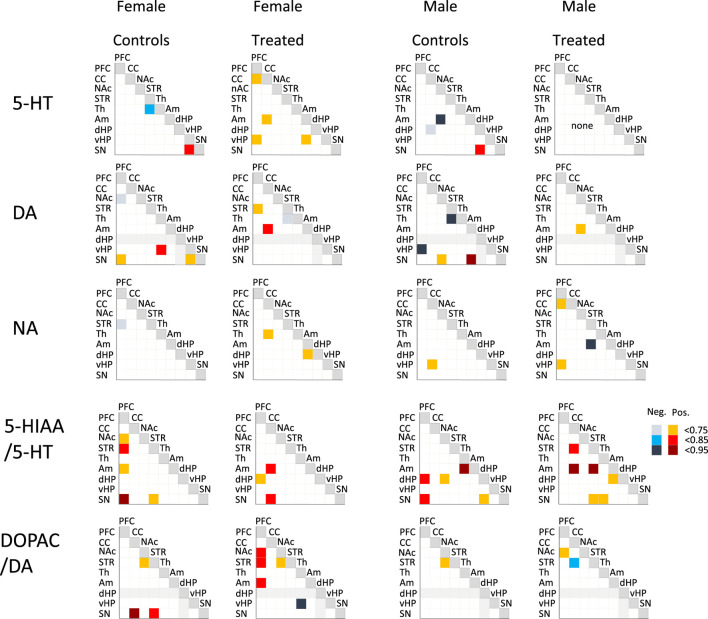
Effect of adolescent alcohol and cannabis exposure in a binge-like pattern combined with daily nicotine administration (P30–P60) on Pearson correlation coefficients of monoamines and turnover ratios across sampled brain regions in male and female Long-Evans rats. Correlations coefficients (r) for pairs of brain regions are reported for serotonin (5-HT), dopamine (DA), noradrenaline (NA), and the turnover ratios 5-HIAA/5-HT and DOPAC/DA when statistically significant (p < 0.05). Correlations are reported for treated (binge-exposed) animals in both sexes PFC, prefrontal cortex; CC, cingulate cortex; NAc, nucleus accumbens; STR, striatum; TH, thalamus; Am, amygdala; dHP, dorsal hippocampus; vHP, ventral hippocampus; SN, substantia nigra. Positive and negative correlations as indicated on the figure. Grey boxes correspond to the absence of correlations due to the limited amount of available data.

Correlational analyses were performed to explore the organization of monoaminergic systems by examining the relationships between monoamines, their metabolites, and turnover ratios both within individual brain regions and across the brain (Figure 6; Supplementary Table S4).

Within single brain regions, correlations between each neurotransmitter and its metabolite were generally stronger in females than in males, particularly for 5-HIAA and 5-HT, while correlations for DA and DOPAC were comparable between sexes. The number of significant correlations observed in control females was lower following binge-like treatment.

When correlations were analyzed across brain regions, the overall number of significant monoaminergic associations was low but displayed distinct sex-dependent patterns. The baseline correlation profiles differed between males and females for all neurochemical indices (Figure 6). Following binge exposure, females and males showed divergent responses. For 5-HT tissue levels, treatment slightly increased inter-regional correlations in females but reduced them in males.

The 5-HIAA to 5-HT ratio revealed correlations involving the PFC and SN in both sexes under control conditions. These associations were reduced in females after treatment and shifted in males toward new correlations involving the amygdala. For DA, binge exposure led to a clear reduction of inter-regional correlations in males. In both sexes, correlations involving the SN that were present in controls disappeared after treatment. In females, DA-related correlations shifted from the SN in controls to the PFC, CC, and NAc following treatment. This reorganization was also reflected in the DOPAC to DA ratio, which displayed increased correlations involving the PFC in treated females. In contrast, males exhibited fewer DA-related correlations overall, although a single correlation between the PFC and NAc emerged after treatment.

NA content displayed minimal inter-regional correlations in controls but showed a slight overall increase in treated animals of both sexes.

**FIGURE 7:**
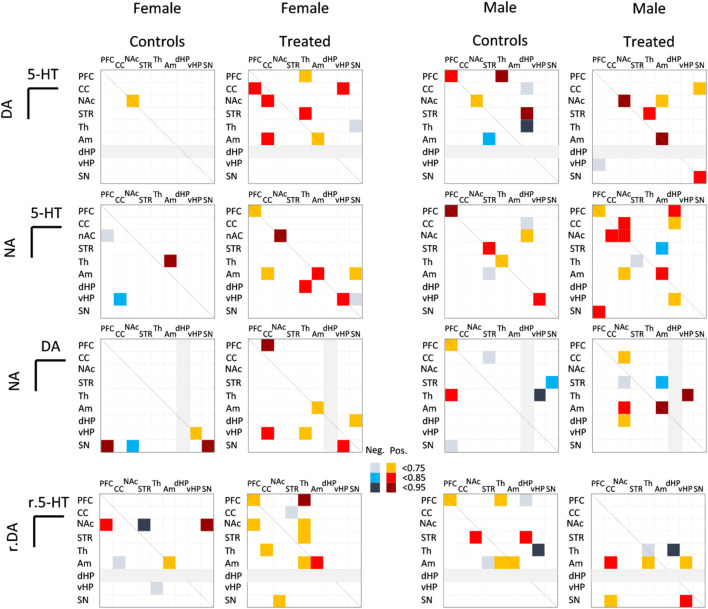
Effect of adolescent alcohol and cannabis exposure in a binge-like pattern combined with daily nicotine administration (P30–P60) on Pearson correlation coefficients of monoamine levels and turnover ratios across sampled brain regions in male and female Long-Evans rats. Correlation coefficients (r) are shown for pairs of brain regions and monoamines (5-HT/DA, 5-HT/NA, DA/NA) or turnover ratios (5-HIAA/5-HT *versus* DOPAC/DA; r.5-HT/r.DA) when significant (p < 0.05). Correlations are reported for treated (binge-exposed) animals in both males and females. PFC, prefrontal cortex; CC, cingulate cortex; NAc, nucleus accumbens; STR, striatum; TH, thalamus; Am, amygdala; dHP, dorsal hippocampus; vHP, ventral hippocampus; SN, substantia nigra. Positive and negative correlations as indicated in the figure. Grey boxes correspond to the absence of correlations due to the limited amount of available data.

When parameters were combined to assess multi-system interactions across the brain (Figure 7), binge-like exposure produced a clear increase in the number of inter-regional correlations in females for all combinations of neurotransmitter pairs (5-HT/DA, NA/5-HT, DA/NA) and turnover indices (5-HIAA/5-HT and DOPAC/DA). In contrast, males exhibited a mixed response, with binge exposure enhancing some relationships (NA/5-HT, DA/NA), leaving others unchanged (5-HT/DA), and reducing those involving turnover ratios. In both sexes, the amygdala consistently emerged as a central node of connectivity, participating in multiple correlations across treatment conditions. In treated females, additional correlations involved the PFC, whereas in males, these associations tended to shift away from the PFC toward the NAc, particularly for NA/DA and 5-HT/DA pairs.

## Discussion

Using behavioral and neurochemical analyses, we show that adolescent binge-like exposure to ethanol and cannabinoids in chronically nicotine-treated Long-Evans rats produces persistent sex-dependent effects in adulthood. Females displayed reduced exploration and increased anxiety-like behavior in the HB test, whereas males showed minimal behavioral changes. No anxiety-like effects were detected in the EPM in either sex. Neurochemically, females exhibited increased serotonergic activity in cortical and hippocampal regions, while males showed reduced 5-HT in the PFC and increased 5-HT in the SN. These alterations were associated with sex-specific reorganization of monoaminergic networks, enhancing cortical-limbic integration in females and disrupting dopaminergic synchrony in males.

### Effect of age and sex in control animals

The present results also revealed age- and sex-dependent effects on behavioral performance in control animals, accompanied by sex-specific differences in monoaminergic levels and their interregional correlations. From P30 to P90, rats showed a progressive decrease in exploratory activity and a concomitant increase in anxiety-like behavior in the HB test. At the pre-treatment time point (P30), corresponding to early adolescence, quantitative sex differences were minimal (e.g., body grooming), consistent with our recent observations in the same rat strain. Similarly, at P60, confirming previous findings (Casarrubea et al., 2024), and P90, quantitative measures continued to show only minimal sex differences. In the EPM, overall open-arm locomotion remained unchanged; however, females at P60 and P90 displayed increased open-arm entries, suggesting reduced anxiety-like behavior, consistent with earlier reports (Imhof et al., 1993; Vendruscolo et al., 2008; Duchesne et al., 2009), though findings vary by strain and paradigm (Beck and Luine, 2002; Knight et al., 2021). These differences may also reflect higher baseline locomotor or metabolic activity in females and the limited validation of traditional anxiety paradigms for female rodents (Johnston and File, 1991).

Sex differences in neurochemical measures under control conditions were generally modest, consistent with previous reports (Beck and Luine, 2002). Tissue levels of 5-HT, DA, NA, and their metabolites were largely similar between sexes across brain regions, except for lower 5-HT in the female PFC. In contrast, other studies in the same strain reported higher DA and 5-HT in the ventral PFC of females, while males showed higher metabolite levels and turnover across several corticolimbic regions (Duchesne et al., 2009).

Correlational analyses of monoamine parameters, which use variability in quantitative measures to probe neurochemical interactions across brain regions (Butler et al., 2025a), revealed clear sex dependent differences in monoaminergic network organization. Females showed stronger correlations between neurotransmitters and their metabolites, particularly 5-HT and 5-HIAA, suggesting tighter coupling of 5-HT availability and metabolism. Inter-regional correlation patterns also differed between sexes, indicating sex dependent organization of monoaminergic networks, consistent with evidence linking these systems to large scale networks such as the default mode network (Bär et al., 2016), that show sex specific differences in information processing (Chen et al., 2024).

### Effect of treatment - behavior

Combined binge-like ethanol and WIN55,212-2 exposure (8 intoxications) exposure with chronic nicotine produced subtle, long-lasting behavioral alterations that were both age- and sex-dependent. At P60, females exhibited reduced locomotor and exploratory activity (climbing, rearing, head-dipping), accompanied by increased grooming and edge-sniffing, a profile consistent with enhanced anxiety-like behavior and neophobia. In contrast, males showed comparatively blunted responses, appearing less sensitive to adolescent exposure. These outcomes differ from our previous work, in which higher-dose nicotine increased anxiety-like behavior in males (Casarrubea et al., 2015; Casarrubea et al., 2020; Casarrubea et al., 2024), whereas adolescent nicotine exposure reduced it in females (Casarrubea et al., 2024). After a 30-day washout (at P90), treated females still displayed reduced rearing and head-dipping, suggesting partial persistence of an anxiety-like phenotype, whereas males remained comparable to controls as in P60. On the other hand, no treatment effects were detected in the EPM, differently from the HB, indicating task-dependent behavioral outcomes.

Previous studies reported that adolescent WIN55,212-2 (1 mg/kg daily) did not alter anxiety-like behavior in male rats in the EPM or open field (Bambico et al., 2010) and intermittent ethanol exposure during adolescence (3 g/kg every 48h, 10 intoxications) produced no significant anxiety-like alterations in either sex (Matthews et al., 2023). Only higher ethanol dose or different exposure window induced an anxiogenic phenotype and these were limited to males at PD 49 without persistence into later life (Towner and Varlinskaya, 2020; Matthews et al., 2023). However, when drugs are combined, as in our protocol, their interaction may produce outcomes that differ from those observed with single compounds, including counteracting effects. For example, chronic adolescent nicotine exposure has been reported to normalize behavioral alterations induced by WIN55,212-2 cannabinoid exposure in mice (Pushkin et al., 2019). Conversely, co-exposure to ethanol and Δ9-tetrahydrocannabinol induces persistent impairments in prefrontal synaptic plasticity that are stronger than those produced by either drug alone (Shi et al., 2024), highlighting the importance of polydrug approaches to better model real-world conditions.

### Effect of treatment - neurochemistry

The effects of polydrug regimen were modest in adulthood and did not alter overall monoamine or metabolite tissue levels. Treatment increased 5-HT and 5-HIAA in the PFC of females and the SN of males, while DA and NA remained unchanged across regions. The 5-HIAA/5-HT ratio, an index of serotonergic turnover (Curzon and Green, 1970), was elevated only in the vHP of treated females, suggesting increased serotonergic activity in this region.

Since alcohol, WIN55,212-2, and nicotine affect monoaminergic systems (Becker and Hu, 2008; Volkow and Blanco, 2023), the lack of major changes in absolute monoamine levels may reflect both counteracting drug interactions and/or allostatic adaptations in neurotransmitter systems (Koob and Le Moal, 2001; Koob, 2008), underlying the observed behavioral effects. Consistent with this, correlation analyses of tissue monoamines, which capture relationships beyond mean ± SEM measures (Butler et al., 2025a), revealed a pronounced sex-dependent reorganization of monoaminergic network interactions.

In females, treatment increased DA correlations with 5-HT and NA, indicating enhanced cross system coupling and reorganization of monoaminergic networks. In contrast, males showed reduced DA correlations, particularly involving the SN, suggesting decreased dopaminergic coherence. In females, DA correlations shifted from the SN to cortical and limbic regions (PFC, CC, NAc), consistent with greater frontolimbic integration linked to reduced exploration and increased anxiety like behavior. This pattern aligns with evidence that chronic nicotine alters VTA DA neuron responsiveness to amygdalar and prefrontal inputs (Dongelmans et al., 2021; Pierucci et al., 2022), suggesting that our observed correlation shifts may reflect functional remodeling of DA neuron connectivity toward regions involved in affective and motivational regulation. Consistent with this view, adolescent co-exposure to ethanol and Δ9-tetrahydrocannabinol has been shown to induce long-lasting alterations in synaptic plasticity in the medial PFC of Long-Evans rats, indicating persistent disruptions of prefrontal circuit function following polydrug exposure (Shi et al., 2024).

The serotonergic system also showed reorganization after binge like polydrug exposure. In control females, strong 5-HT/5-HIAA correlations were reduced after treatment, suggesting partial decoupling of 5-HT synthesis and metabolism, as seen in adaptive responses to chronic pharmacological challenges (Sánchez and Hyttel, 1999; Tassin, 2008). Across regions, 5-HT correlations slightly increased in treated females but decreased in males, supporting sex divergent serotonergic adaptations (Müller and Homberg, 2015). Analysis of the 5-HIAA/5-HT ratio revealed altered connectivity involving the PFC and SN in both sexes, with reduced coupling in females and new amygdala related interactions in males. This reorganization is consistent with evidence that serotonergic modulation of corticolimbic circuits, particularly the PFC and amygdala, is dynamically regulated by chronic drug exposure and DA-5-HT cross talk (Lanteri et al., 2008, Di Giovanni et al., 010; Celada et al., 2013). Overall, these findings suggest that polydrug exposure reorganizes monoaminergic network communication, with the amygdala acting as a key integrative node and the PFC more involved in females. Females showed a global increase in correlations after binge exposure, whereas males displayed mixed changes. These sex specific rearrangements involving the amygdala, PFC, and NAc support the concept of monoaminergic allostasis (Koob and Le Moal, 2001), where chronic drug exposure maintains neurotransmitter homeostasis while establishing new inter regional regulation. The persistence of these correlation changes without major monoamine alterations indicates adaptive plasticity rather than overt neurochemical dysregulation (Fuxe et al., 1990; Koob and Le Moal, 2001; Koob, 2008).

### Methodological considerations and limitations

Repeated testing can affect novelty driven behaviors through habituation or one trial tolerance (File and Wardill, 1975; Carobrez and Bertoglio, 2005; Walf and Frye, 2007), the long inter-test interval (30 days) and the inclusion of age and sex matched control groups tested at identical time points likely minimized these effects (Schrader et al., 2018). Moreover, repeated gavage administration may also have induced mild procedural stress, potentially reducing baseline sex differences, particularly in females (Luine et al., 2007), and engaging monoaminergic circuits involved in stress related allostatic adaptations (Koob and Le Moal, 2001; Koob, 2008).

The behavioral correlates of the polydrug protocol observed here are subtle. One possible explanation is that they are not readily detectable using conventional quantitative analyses, thus requiring more sensitive multivariate approaches. Indeed, multivariate T-pattern analysis, a behavioral approach that detects hidden, recurring sequences of events (Casarrubea et al., 2023) has revealed sex differences in the emotional state of rats that were not detected by standard quantitative measures, both in naïve animals at P30 (Casarrubea et al., 2025) and after adolescent nicotine exposure at P60 (Casarrubea et al., 2024). This interpretation is consistent with previous findings demonstrating alterations in reward and memory following this adolescent polydrug paradigm, particularly in females, and in males only under higher cognitive demand conditions (Abela et al., 2023).

The absence of effects of the polydrug treatment in the EPM likely reflects task-specific sensitivity, as previously mentioned. The EPM primarily measures approach–avoidance conflict in a highly anxiogenic context (Walf and Frye, 2007), whereas the HB is more sensitive to spontaneous exploration, neophobia, and risk assessment (Brown and Nemes, 2008; Casarrubea et al., 2023). Consistent with this distinction, WIN55,212-2 produced anxiolytic-like effects in the HB but not in the EPM (Cassar et al., 2022; De Deurwaerdère et al., 2022), and adolescent methylphenidate exposure altered open-field behavior without affecting EPM performance (Vendruscolo et al., 2008).

### Translational implications

Despite these limitations, the present findings suggest that even relatively mild patterns of adolescent recreational drug use, including nicotine, alcohol, and cannabis, may produce long lasting and sex specific alterations in brain function. Intermittent polydrug exposure during this developmental period appears sufficient to promote anxiety like behavior and to reorganize monoaminergic networks involved in emotional regulation, as well as motivational and cognitive processes (Abela et al., 2023). Consistent with this, human studies indicate that combined exposure to these substances during adolescence or emerging adulthood is associated with alterations in hippocampal structure and function, with females often showing greater vulnerability (Harper et al., 2023). Together, these observations emphasize the importance of early prevention strategies (Schlienz and Lee, 2018) and the need to consider sex-specific vulnerability to the long-term consequences of adolescent drug use.

## Data Availability

The raw data supporting the conclusions of this article will be made available by the authors, without undue reservation.
