# Supplementary material for: Sex-specific behavioral and monoaminergic network alterations following adolescent binge-like ethanol and WIN55,212-2 exposure under chronic nicotine in rats
Source: Front Pharmacol. 2026 Apr 10;17:1798364. doi: 10.3389/fphar.2026.1798364 (PMC13106439; doi:10.3389/fphar.2026.1798364)
Supplement: Supplementary file 1 [file DataSheet1.pdf]

Supplementary Files

Sex-Specific Behavioral and Monoaminergic Network Alterations Following Adolescent Binge-like Ethanol and WIN55,212-2 Exposure Under Chronic Nicotine in Rats

Philippe De Deurwaerdère[1,2], Katie Haywood[3], Norbert Abela[3], Maurizio Casarrubea[4], Giuseppe Di Giovanni[3,5,6,7]

Supplementary Table 1: Effect of binging on the metabolism of DA and 5-HT in the selected brain regions of long-Evans female and male rats.

Supplementary Table 2: Effect of binging on the metabolism of DA and 5-HT in the selected brain regions of long-Evans female and male rats.

Supplementary Table 3: Statistical report of binge on tissue levels of various compounds in the selected brain regions of long-Evans female and male rats.

Supplementary Table 4: Effect of binging on the correlative link for a monoamine and its metabolite in a single brain region in long-Evans female and male rats.

Supplementary Table S1. Three-way mixed ANOVA results for Hole-Board (HB) and Elevated Plus Maze (EPM)

**Behavioral Test Variable Effect F (df) / p value**
HB (Frequency) Walking frequency T × S F(1,36) = 14.83; p = 0.0005
HB (Frequency) Walking frequency A F(2,36) = 6.90; p = 0.0018
HB (Frequency) Walking frequency A × T F(2,36) = 3.35; p = 0.0406
HB (Frequency) Walking frequency A × T × S F(2,36) = 4.30; p = 0.0172
HB (Frequency) Face grooming frequency A F(2,36) = 19.77; p < 0.0001
HB (Frequency) Body grooming frequency S F(1,36) = 6.37; p = 0.0162
HB (Frequency) Body grooming frequency A F(2,36) = 5.80; p = 0.0046
HB (Frequency) Head dip frequency T F(1,36) = 4.27; p = 0.0460
HB (Frequency) Head dip frequency A F(2,36) = 62.88; p < 0.0001
HB (Frequency) Head dip frequency A × S F(2,36) = 3.24; p = 0.0448
HB (Duration) Face grooming duration A F(2,36) = 22.01; p < 0.0001
HB (Duration) Face grooming duration A × T F(2,36) = 3.66; p = 0.0307
HB (Duration) Climbing duration T F(1,36) = 7.88; p = 0.0080
HB (Duration) Climbing duration A × S F(2,36) = 5.91; p = 0.0042
HB (Duration) Rearing duration T F(1,36) = 10.88; p = 0.0031
HB (Duration) Rearing duration A F(2,36) = 71.80; p < 0.0001
HB (Duration) Edge sniffing duration T F(1,36) = 13.86; p = 0.0007
HB (Duration) Edge sniffing duration A F(2,36) = 26.21; p < 0.0001
HB (Duration) Edge sniffing duration A × S F(2,36) = 6.04; p = 0.0038
EPM Open arm time (%) A F(2,36) = 21.59; p < 0.0001
EPM Open arm entries (%) S F(1,36) = 6.64; p = 0.0142
EPM Open arm entries (%) A F(2,36) = 30.89; p < 0.0001
EPM Open arm entries (%) A × T F(2,36) = 5.51; p = 0.0059
EPM Total distance travelled T F(1,36) = 0.26; p = 0.6145
EPM Total distance travelled S F(1,36) = 0.76; p = 0.3892
EPM Total distance travelled A F(2,36) = 1.31; p = 0.2768

Summary of ANOVA results for behavioral parameters measured in the Hole-Board (HB) and Elevated Plus Maze (EPM) tests. The table reports F values with corresponding degrees of freedom (df) and p values for main effects and interactions. For HB and EPM behavioral data, age (A: P30, P60, P90) was included as a within-subject factor, while treatment (T: control vs polydrug exposure) and sex (S: male vs female) were between-subject factors. Only statistically significant effects ($p < 0.05$) are shown, except for total distance travelled in the EPM, which is reported to document the absence of locomotor differences. Frequency and duration measures are presented separately for HB variables.

Supplementary Table 2: Effect of binging on the metabolism of DA and 5-HT in the selected brain regions of long-Evans female and male rats.

	Female		Male	
	Control	treated	Control	treated
5-HIAA				
Prefrontal cortex	284 ± 74	456 ± 35**	342 ± 42	325 ± 34^^
Cingulate Cortex	322 ± 32	303 ± 18	264 ± 28	275 ± 27
Nucleus accumbens	271 ± 21	286 ± 27	257 ± 23	278 ± 33
Striatum	414 ± 44	504 ± 25	386 ± 42	377 ± 26
Thalamus	653 ± 55	755 ± 26	558 ± 48	595 ± 41^
Amygdala	395 ± 35	537 ± 59	423 ± 33	439 ± 27
Dorsal hippocampus	417 ± 34	449 ± 24	380 ± 36	390 ± 35
Ventral hippocampus	508 ± 31	700 ± 54***	458 ± 33	535 ± 24^^
Substantia nigra	641 ± 46	760 ± 102	624 ± 65	689 ± 72
5-HIAA/5-HT				
Prefrontal cortex	4 ± 0.55	3.34 ± 0.36	2.67 ± 0.25	3.22 ± 0.45
Cingulate Cortex	1.6 ± 0.12	1.5 ± 0.16	1.3 ± 0.11	1.43 ± 0.18
Nucleus accumbens	1.19 ± 0.1	0.93 ± 0.1	1 ± 0.08	1 ± 0.06
Striatum	1.3 ± 0.06	1.24 ± 0.03	1.16 ± 0.12	1.23 ± 0.06
Thalamus	2.78 ± 0.44	2.45 ± 0.4	2.33 ± 0.53	2.24 ± 0.36
Amygdala	1.58 ± 0.16	1.98 ± 0.16	1.8 ± 0.28	1.76 ± 0.15
Dorsal hippocampus	3.28 ± 0.18	3.3 ± 0.23	2.8 ± 0.44	3 ± 0.33
Ventral hippocampus	3.1 ± 0.21	4.1 ± 0.4*	2.5 ± 0.4	3.23 ± 0.21
Substantia nigra	1.18 ± 0.06	1.46 ± 0.15	1.21 ± 0.19	0.95 ± 0.08
DOPAC				
Prefrontal cortex	28.3 ± 5.1	33.3 ± 4.2	33.6 ± 4.8	27.7 ± 4.2
Cingulate Cortex	19.1 ± 8.5	14.4 ± 4.6	12.7 ± 2.3	19.9 ± 3.4
Nucleus accumbens	607 ± 73	603 ± 88	625 ± 83	616 ± 68
Striatum	725 ± 90	705 ± 43	628 ± 85	618 ± 17
Thalamus	181 ± 42	85 ± 14.9	95 ± 25	82 ± 12.2
Amygdala	43.9 ± 7.8	75.6± 17	60.4 ± 17	70.3 ± 20.7
Dorsal hippocampus	4.9 ± 0.7	5.9 ± 1.8	7.1 ± 3.5	4.9 ± 0.7
Ventral hippocampus	6.6 ± 1.3	10.6 ± 1.8	6.2 ± 0.8	9.7 ± 0.6
Substantia nigra	108 ± 15.6	111 ± 25	113 ± 18.7	119 ± 17
DOPAC/DA				
Prefrontal cortex	0.66 ± 0.13	0.58 ± 0.1	0.51 ± 0.07	0.5 ± 0.11
Cingulate Cortex	0.42 ± 0.2	0.4 ± 0.13	0.26 ± 0.05	0.51 ± 0.13
Nucleus accumbens	0.25 ± 0.03	0.29 ± 0.03	0.31 ± 0.08	0.25 ± 0.02
Striatum	0.11 ± 0.007	0.1 ± 0.007	0.09 ± 0.1	0.1 ± 0.004
Thalamus	0.33 ± 0.04	0.66 ± 0.37	0.27 ± 0.03	0.4 ± 0.15
Amygdala	0.22 ± 0.02	0.28 ± 0.04	0.27 ± 0.02	0.3 ± 0.06
Dorsal hippocampus[a]	*0.41 ± 0.1*	*0.5 ± 0.04*	*0.5 ± 0.07*	*0.59 ± 0.06*
Ventral hippocampus	0.76 ± 0.2	1.12 ± 0.34	0.46 ± 0.06	1.49 ± 0.32
Substantia nigra	0.29 ± 0.03	0.41 ± 0.07	0.27 ± 0.02	0.26 ± 0.02

*$p<0.05$, **$p<0.01$ with respect to respective control; ^ $p<0.05$, ^^$p<0.01$ between treated groups (PLSD test) [a]n=3-5

Supplementary Table 3: Statistical report of polydrug treatment on tissue levels of various compounds in the selected brain regions of female and male Long-Evans rats.

	F (two-way - ANOVA)						
Region	NA	DA	5-HT	DOPAC	5-HIAA	DOPAC/DA	5-HIAA/5-HT
PFC	(1,33) 2.1 (ns)	(1,32) 0.11 (ns)	(1,33) 9.45**	(1,31) 1.42 (ns)	(1,32) 5.6*	(1,31) 0.12 (ns)	(1,32) H=4.6 ns
CC	(1,33) 3.6 (ns)	(1,33) 0.05 (ns)	(1,33) 0.05 (ns)	(1,32) 1.17 (ns)	(1,33) 0.3 (ns)	(1,32) 0.82 (ns)	(1,33) 0.6 (ns)
NAc	(1,33) 0.01 (ns)	(1,33) 0.85 (ns)	(1,33) 0.47 (ns)	(1,33) 0.001 (ns)	(1,33) 0.007 (ns)	(1,33) 1.32 (ns)	(1,33) 1.15 (ns)
STR	(1,33) 0.04 (ns)	(1,33) 0.09 (ns)	(1,32) 3.42 (ns)	(1,33) 0.005 (ns)	(1,33) 1.96 (ns)	(1,33) 0.01 (ns)	(1,32) H=1.5 ns
TH	(1,32) 1.37 (ns)	(1,32) 1.87 (ns)	(1,32) 1.43 (ns)	(1,32) H=5.9 (ns)	(1,32) 0.6 (ns)	(1,32) 0.19 (ns)	(1,32) H=1.3
Am	(1,32) 0.04 (ns)	(1,30) 0.81 (ns)	(1,32) 0.14 (ns)	(1,32) 0.41 (ns)	(1,32) 2.11 (ns)	(1,32) 0.76 (ns)	(1,32) 1.32 (ns)
dHP	(1,32) 0.007*	(3,16) 0.4 (ns)	(1,32) 1.57 (ns)	(1,26) 0.59 (ns)	(1,32) 0.12 (ns)	(3,16) 0.9 (ns)	(1,32) 0.18 (ns)
vHP	(1,33) 0.12 (ns)	(1,27) 1.73 (ns)	(1,33) 2 (ns)	(1,26) H=6.8 (ns)	(1,33) 7.5***	(1,21) H=8.34*	(1,33) H=6.9 (ns)
SN	(1,33) 1.76 (ns)	(1,32) 0.9 (ns)	(1,32) 4.13 (=0.05)	(1,32) 0.006 (ns)	(1,33) 0.1 (ns)	(1,32) 1.9 (ns)	(1,33) H=9.8*

The two-way ANOVA was substituted by the Kruskall-Wallis analysis when the data were non parametric. For that reason, we report the H factor of the Kruskall-Wallis for a few analyses. PFC, prefrontal cortex; CC, cingulate cortex; NAc, nucleus accumbens; STR, striatum; TH, thalamus; Am, amygdala; dHP, dorsal hippocampus; vHP, ventral hippocampus; SN, substantia nigra. * p<0.05; **p<0.01; p<0.001.

Supplementary Table 4: Effect of binging on the correlative link for a monoamine and its metabolite in a single brain region in long-Evans female and male rats.

	**DOPAC vs DA**	**5-HIAA vs 5-HT**
**Female - saline**		
Prefrontal Cortex	P<0.05	P<0.001
Cingulate cortex	ns	P<0.05
Thalamus	P<0.05	ns
Nucleus accumbens	ns	P<0.01
Striatum	P<0.001	P<0.05
Substantia nigra	P<0.05	P<0.001
Amygdala	P<0.05	P<0.05
Dorsal hippocampus	NA	P<0.05
Ventral hippocampus	ns	P<0.05
**Female - Treated**		
Prefrontal Cortex	ns	ns
Cingulate cortex	P<0.05	ns
Thalamus	P<0.01	ns
Nucleus accumbens	P<0.05	ns
Striatum	ns	P<0.001
Substantia nigra	P<0.01	P<0.01
Amygdala	P<0.001	P<0.001
Dorsal hippocampus	NA	ns
Ventral hippocampus	ns	ns
**Male- saline**		
Prefrontal Cortex	ns	P<0.05
Cingulate cortex	ns	ns
Thalamus	P<0.001	ns
Nucleus accumbens	P<0.01	P<0.01
Striatum	ns	P<0.05
Substantia nigra	P<0.001	ns
Amygdala	P<0.05	ns
Dorsal hippocampus	NA	ns
Ventral hippocampus	P<0.05	ns
**Male - Treated**		
Prefrontal Cortex	ns	ns
Cingulate cortex	ns	ns
Thalamus	P<0.001	ns
Nucleus accumbens	P<0.01	P<0.001
Striatum	ns	P<0.05
Substantia nigra	P<0.001	ns
Amygdala	P<0.001	ns
Dorsal hippocampus	NA	ns
Ventral hippocampus	ns	ns

Using the correlations of Pearson, the table reports the significance of the correlations for DA and its metabolite DOPAC and 5-HT and its metabolite 5-HIAA within a single brain region. The significance of the correlation is reported as indicated; ns, non significant; NA, not available
